# Development of Suitable Solvent System for Downstream Processing of Biopolymer Pullulan Using Response Surface Methodology

**DOI:** 10.1371/journal.pone.0077071

**Published:** 2013-10-15

**Authors:** Anirban Roy Choudhury, Paramita Bhattacharjee, Gandham S. Prasad

**Affiliations:** 1 CSIR-Institute of Microbial Technology, Council of Scientific and Industrial Research (CSIR), Chandigarh, India; 2 Department of Food Technology & Bio-Chemical Engineering, Jadavpur University, Kolkata, India; Oak Ridge National Laboratory, United States of America

## Abstract

Downstream processing is an important aspect of all biotechnological processes and has significant implications on quality and yield of the final product. Several solvents were examined for their efficacy on pullulan precipitation from fermentation broth. Interactions among four selected solvents and their effect on pullulan yield were studied using response surface methodology. A polynomial model was developed using D-optimal design and three contour plots were generated by performing 20 different experiments and the model was validated by performing optimization experiments. The results indicated that lower concentration of ethanol in combination with the other three solvents has resulted in higher yield of polymer from fermentation broth and the optimized solvent system was able to recover 1.44 times more pullulan as compared to the conventional ethanolic precipitation method. These observations may help in enhancing efficiency of pullulan recovery from fermentation broth and also result in reduced cost of production for the final product.

## Introduction

 Pullulan is one of the widely studied polysaccharides among various α- linked glucans produced by fungi and was first reported by Bauer in 1938 [1]. This is a neutral, water soluble, homopolymer of maltotriose subunits, linked by both α-1,4 and α-1,6 glycosidic linkages. The α-1,4 and α-1,6 linkages in this polymer are uniquely altered in a ratio of 2:1. This unique linkage pattern has rendered the polymer significant physico-chemical properties like structural flexibility, easy derivability, enhanced solubility etc [2,3]. The biopolymer is a potential candidate for application in several industrial sectors like food, pharmaceuticals, cosmetics, biomedical etc. [4,5]. Recently, this polymer has also become popular in the area of drug and gene delivery [6,7].

*Aureobasidium pullulans*, a polymorphic fungus, has mostly been exploited for fermentative production of pullulan [8].The comparatively higher price of commercially available pullulan is due to the difficulties associated with its fermentation process which include the formation of melanin-like pigment, decrease in molecular weight of the polymer as the fermentation proceeds and high viscosity of the broth due to production of pullulan[9,10]. Product recovery is an important aspect in all the biotechnological processes. Although, strategies of purification depend on desired use of the final product and in most cases several different processes like cell separation, solvent extraction, decolorization, chromatographic purification etc. are involved in purification of fermentation products. All these processes have significant effect on the cost of the final product and may even account for more than 60% of total cost for production [11]. Therefore, development of a simplified and cost effective downstream process for microbial polysaccharides is a major challenge for their commercialization. There are several reports on upstream processing of pullulan viz. optimization of carbon sources and other media components and operating conditions like pH, temperature, agitation and aeration during fermentation [3,12]. However, studies on downstream processing of pullulan from cell free fermentation broth are very much limited. In most cases pullulan is recovered from fermentation broth using solvent precipitation techniques where the polysaccharide is precipitated using ethanol and further purified by dialysis. Recently, Kacchwa et al. [13] reported that combination of more than one solvent may result in better yield during solvent precipitation of pullulan from fermentation broth. However, there were no reports of optimized solvent extraction process coupled with suitable combination of different solvents.

Classical one point optimization techniques may not be suitable while optimizing pullulan recovery from fermentation broth and this also may not be able to explain the effect of interaction among different solvents on pullulan recovery. Statistical experimental design techniques are very useful tools when more than one factor is studied at a time. These statistical models help in understanding the interactions among the different variables at various levels and in calculating the optimal level of each variable [14].

 Response surface methodology (RSM) is one such statistical method which can be employed for this purpose and may be used for understanding interactions among several factors in a multi-factor system [15]. RSM is a collection of statistical and mathematical techniques useful for developing, improving, and optimizing processes in which response(s) of interest is influenced by several variables and the objective is to understand the effect of simultaneous changes in the variables on the response(s). It has important applications in the design, development and formulation of new products, as well as in the improvement of existing product design and defining the effect(s) of the independent variables, alone or in combinations, on the processes. In addition to analyzing the effects of the independent variables, this experimental methodology fits the responses in a suitable mathematical model which accurately describes chemical or biochemical processes [16]. As a powerful statistical and mathematical tool, RSM provides important information regarding the optimal level of each variable along with its interaction with other variables and their influence on production and has been used extensively for optimization of several fermentation processes [17]. Recently, we have reported the effect of interaction among media components on pullulan elaboration using the same technique [18]. Earlier, Chen et. al. [19] have also used RSM for optimization of media components for pullulan production. However, use of RSM for developing a suitable downstream process in biotechnology based products is very limited. In the present study, various organic solvents were screened for recovering pullulan from fermented broth. Then RSM was applied by taking selected solvents as independent variables to understand the effect of their interaction on pullulan recovery. This is the first report of application of RSM to enhance yield during recovery of pullulan from fermentation broth.

## Materials and Methods

### Ethics Statement

The samples were taken from flowers (*Caesulia axillaris*) grown in the roadside and it was not a protected land or private property. Hence this does not require any permission for such activities and this flower also does not come under “endangered or protected species".

### 1: Materials

*Aureobasidium pullulans* RBF 4A3 used in present study was isolated from flowers of *Caesulia axillaris* from Rawatbhata, Rajasthan, India. The organism was maintained on YPD agar plates at 4°C and 10% glycerol stocks were made and stored at -80°C for long term preservation [20]. 

Media components such as yeast extract, peptone, dextrose used for growing the fungus were purchased from M/S Himedia, Mumbai, India and solvents used in downstream processing were obtained from M/S Merck India Ltd, Mumbai, India.

### 2: Methods

#### 2.1: Media

The inoculum was grown in YPD medium and production medium contained 1.5% (w/v) yeast extract, 2% (w/v) peptone, 15.5% (w/v) dextrose and distilled water.

#### 2.2: Fermentation conditions

The inoculum was grown by inoculating 50 ml of medium in a 250 ml conical flask using a fresh plate culture grown on YPD agar medium with subsequent incubation for 24 hours at 28°C with an agitation speed of 200 rpm (Innova 4230, New Brunswick Scientific). Pullulan fermentation was carried out in 250 ml Erlenmeyer flasks containing 50 ml production medium which was inoculated with 2.5 ml of inoculum at 28°C for 96 h with constant shaking at 200 rpm (Innova 4230, New Brunswick Scientific).

#### 2.3: Harvesting

After completion of fermentation, cells were separated by centrifugation (Sigma laboratory centrifuge, 6K15) at a speed of 10,000 rpm for 20 min, followed by filtration through 0.2 micron filters. The supernatant obtained was precipitated with selected organic solvent(s) and the precipitated exopolysaccharide (EPS) was separated by centrifugation at 10,000 rpm for 20 minutes. This precipitate was re-dissolved in de-ionized water and dialyzed for 48 hours to remove small molecular impurities from it. EPS was re-precipitated using organic solvent and dried in oven at 80°C for overnight and weighed. The pullulan content in the precipitate was determined by the method as described by Sharma et.al. [21] and was expressed as pullulan percentage. Further, the amount of pullulan produced was calculated by multiplying the EPS produced by pullulan percentage and reported as “g/L pullulan produced”.

#### 2.4: Screening of organic solvents

Eight different organic solvents, namely ethanol, acetone, isopropanol (IPA), tetra hydro furan (THF), diethyl ether, butanol, methanol and hexane were screened for pullulan precipitation from cell free fermented broth. In each case, cell free broth was extracted with organic solvent in ratios ranging from 1:1 to 1:7(v/v). EPS was precipitated and pullulan was measured as per the method described above. All the experiments were carried out in triplicate and the averages of the data obtained are reported. Standard deviation was calculated in each case and reported along with the data to ensure statistical reliability of the data obtained.

### 3: Experimental Design and Statistical Analysis

Based on the observations made during screening experiments, four solvents viz. ethanol, acetone, IPA and THF were selected for further study. RSM was used to understand the effect of interaction among these solvents and their contribution in yield of pullulan during solvent extraction process. A four factor three level D-optimal design was employed for the purpose. While designing the experiments ethanol, acetone, IPA and THF were considered as four independent variables and designated as A, B, C and D respectively. The experimental ranges and restrictions imposed on the ratio of the selected solvents are shown in Table 1. These independent variables were varied at 3 levels which were coded as -1, 0 and +1. The model was developed as a matrix which included centre points, replicates, vertical axis points, centre edge points and axial points to obtain a wholesome response throughout the matrix. This resulted into a set of 20 experiments which include experiments for 1 center point, 10 model points, 4 replicates and 5 more to estimate the lack of fit in the model (Table 2). Experiments were performed thrice for each run and average of the data obtained was considered as response for each run. Values of standard deviations were also calculated in each case to confirm reproducibility of the experimental data. In each case, the amount of pullulan produced was measured as described earlier. Multiple regression analysis of the experimental data obtained was performed using Design expert (ver. 8.0.7.1) software. A special cubic model was developed by analyzing the data and the model is being represented by following equation

**Table 1:** Experimental ranges for independent variables and constraints.

***Factors***	***Experimentalrange***		***Constraints***
	***Low value(ml)***	***High value(ml)***	
A	0	25	A+B+C+D=25
B	0	25	A+B+C+D=25
C	0	25	A+B+C+D=25
D	0	25	A+B+C+D=25

**Table 2:** Experimental data obtained from D-Optimal RSM studies to understand the effect of interaction among organic solvents on precipitation of EPS.

***Std***	***Run***	***Ethanol***	***Acetone***	***IPA***	***THF***	***Response***
		***(ml)***	***(ml)***	***(ml)***	***(ml)***	***EPS (g/L)***
6	1	25.000	0.000	0.000	0.000	50.53±0.02
12	2	15.625	3.125	3.125	3.125	49.28±0.08
14	3	3.125	3.125	15.625	3.125	54.55±0.12
3	4	12.500	0.000	12.500	0.000	50.99±0.25
8	5	0.000	0.000	25.000	0.000	56.32±0.25
5	6	0.000	0.000	12.500	12.500	53.81±0.25
9	7	0.000	0.000	0.000	25.000	61.48±0.05
7	8	0.000	25.000	0.000	0.000	60.94±0.21
19	9	0.000	0.000	0.000	25.000	61.16±0.12
18	10	0.000	0.000	25.000	0.000	56.76±0.17
4	11	0.000	12.500	0.000	12.500	54.68±0.12
15	12	3.125	3.125	3.125	15.625	57.07±0.21
11	13	6.250	6.250	6.250	6.250	51.26±0.17
2	14	12.500	0.000	0.000	12.500	60.57±0.12
13	15	3.125	15.625	3.125	3.125	53.65±0.12
17	16	0.000	25.000	0.000	0.000	60.01±0.17
1	17	0.000	12.500	12.500	0.000	56.32±0.21
20	18	12.500	12.500	0.000	0.000	52.28±0.21
16	19	25.000	0.000	0.000	0.000	52.05±0.09
10	20	12.500	12.500	0.000	0.000	52.24±0.29

Y=a_o_+a_1_A+a_2_B+a_3_C+a_4_D+a_5_AB+a_6_AC+a_7_AD+a_8_BC+a_9_BD+a_10_CD+a_11_ABC+a_12_ABD+a_13_BCD

where Y is the predictive response, and A to D represents independent variables as mentioned earlier and interaction between individual factors are indicated by combination factors (e.g. AB, ABC etc). The regression model is rotatable and can be used to understand and measure the response of interaction among the individual independent variables within the design space. The values of responses obtained by performing experiments as suggested by the model were statistically evaluated using ANOVA and significance of the model was determined by calculating Fischer’s test value (F value) and fitness of the data obtained was evaluated using R^2^. The model was represented by a contour plots generated by using the data obtained with the help of Design Expert software. These contour plots were used to navigate the design space and obtain a suitable composition of solvents which will result in higher recovery of pullulan from fermentation broth. The model was validated by performing all experiments in triplicate.

##  Results and Discussion

### 1: Screening of organic solvents

Among the eight different solvents used for precipitation of pullulan from fermentation broth, addition of diethyl ether, butanol and hexane in fermentation broth did not result in significant precipitation of the polymer. Hence, acetone, ethanol, THF, IPA and methanol were used for further experiments. The ratios of fermentation broth to solvent were varied from 1:1 to 1:7(v/v) to understand the effect of solvent concentration on pullulan recovery. It was observed that initially pullulan precipitation increased with increase in broth to solvent ratio, however, in all cases once the broth to solvent ratio reached 1:5 (v/v), there was no significant increase in the polymer precipitation, indicating that 1:5 (v/v) is the optimum ratio for solvent precipitation of pullulan using selected solvents (Table 3). Although some of the earlier reports [13] indicated that use of more than one solvent in combination may result in higher recovery of pullulan from fermentation broth, there are no studies for understanding the effect of interaction among of different solvents on pullulan recovery. Therefore, it was felt that statistical methods like RSM might be useful for this purpose. Among the screened solvents, pullulan recovery was comparatively low when methanol was used for precipitation. Hence, RSM was applied for understanding the effect of interaction among other four solvents namely ethanol, acetone, IPA and THF on pullulan recovery and a D-optimal design was selected for the purpose.

**Table 3:** Screening of solvents for pullulan precipitation.

Broth: Solvent	Pullulan produced (g/L)				
	Acetone	Ethanol	IPA	THF	Methanol
1:1	38±0.17	40±0.08	39±0.12	40.8±0.22	33±0.21
1:2	45.6±0.12	45.4±0.12	49.2±0.14	47.3±0.21	40±0.21
1:3	53±0.22	47.6±0.25	52.4±0.21	55.7±0.12	40.3±0.16
1:4	60.1±0.12	50.6±0.26	57.4±0.12	59.1±0.25	42±0.25
1:5	59±0.17	51.2±0.22	57.2±0.17	60.8±0.21	44±0.12
1:6	60.3±0.16	49.6±0.21	56.9±0.12	61.2±0.12	44.6±0.17
1:7	60.2±0.17	48.1±0.17	57.6±0.17	60.3±0.17	43.5±0.12

### 2: D-optimal design

A four factor Response surface D-optimal design was used for understanding the effect of interaction of four different solvents on pullulan precipitation from fermentation broth. The ratio of solvents to broth was varied in different levels and the results obtained from the experiments performed as suggested by the developed model are presented in Table 2.

The evaluation of model indicated that this model has 9 degrees of freedom and has 5 degrees of freedom for both lack of fit and pure error. It is recommended to have a minimum 3 degrees of freedom for lack of fit and 4 degrees of feedom for pure error. Hence, the result clearly indicated that the lack of fit test is valid and may be used for evaluation of the model.

The model was tested by using Fischer’s test method for analysis of variance (ANOVA) and the results obtained from ANOVA are presented in Table 4.The ANOVA indicated that interaction among A, C and D was insignificant ( a higher p value ) and hence the term was removed for simplification and better fitting of the model. All other terms have a lower p value and hence those interactions were considered while developing the model. The simplified polynomial equation for pullulan recovery (Y) when expressed in terms of actual factors is:

**Table 4:** Analysis of variance (ANOVA) for all the terms of model.

**Source**	**Sum of Squares**	**Degrees of freedom (df)**	**Mean squre**	**F value**	**p-value prob>F**
Model	288.50	12	24.04	85.54	<0.0001
Linear mixture	151.85	3	50.62	180.10	<0.0001
AB	17.44	1	17.44	62.06	0.0001
AC	6.68	1	6.68	23.78	0.0018
AD	15.31	1	15.31	54.46	0.0002
BC	3.81	1	3.81	13.55	0.0079
BD	30.98	1	30.98	110.23	<0.0001
CD	20.88	1	20.88	74.28	<0.0001
ABC	2.61	1	2.61	9.29	0.0186
ABD	5.56	1	5.56	19.78	0.0030
BCD	8.09	7	8.09	28.79	0.0010
Residual	1.97	2	0.28		
Lack of fit	0.23		0.12	0.33	0.7319
Pure error	1.74	5	0.35		
Cor total	290.47	19			

**Figure 1:**
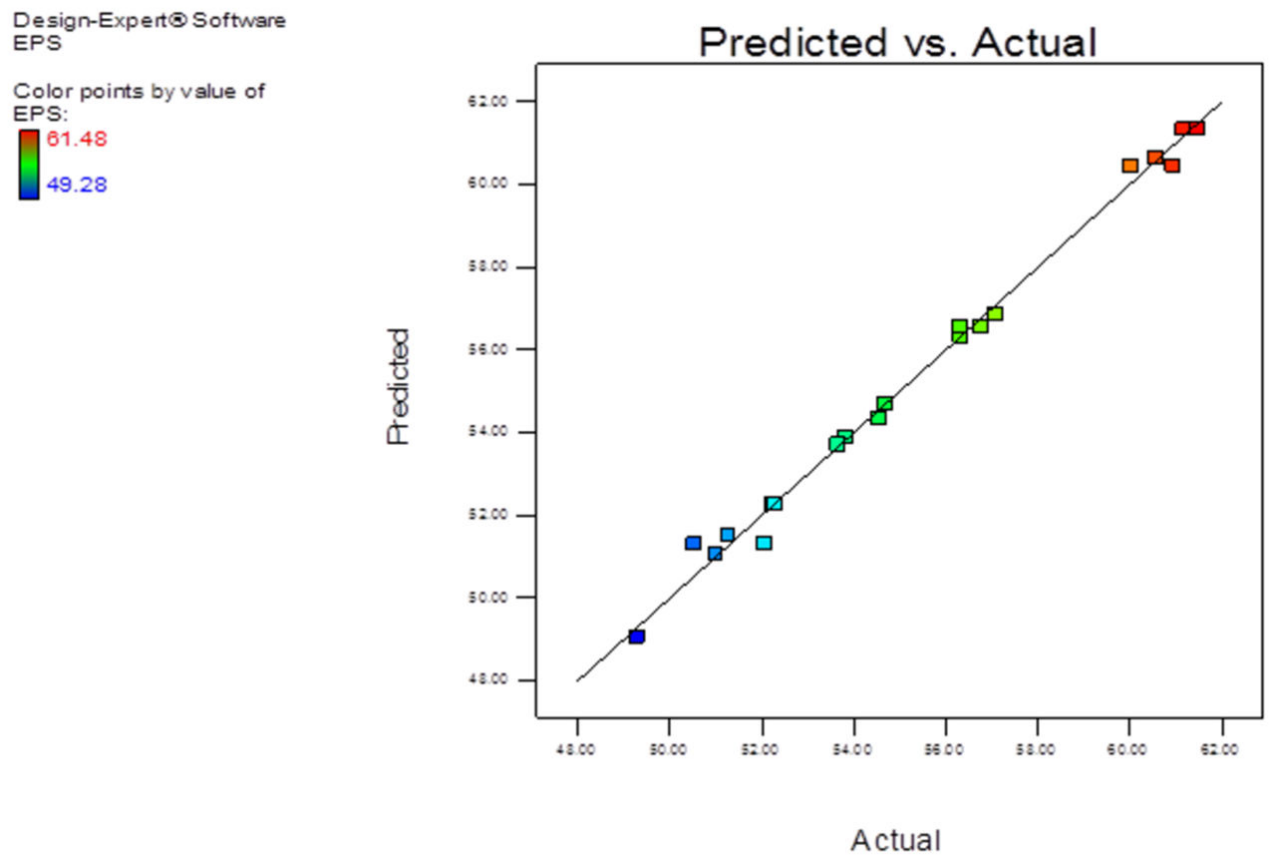
Parity plot: showing the relation between actual and predicted values for pullulan recovery.

*Y=2.05A+2.42B+2.26C+2.45D-0.02AB-0.02AC+0.03AD-0.01BC-0.04BD-0.03D-0.01ABC-0.02ABD+0.02BCD*,

where A, B, C and D are ethanol, acetone, IPA and THF respectively. The F-value of the model was 85.54 which indicated that the model is significant and there might be only 0.01% chance of obtaining such large F-value due to noise. The lack of fit value (0.33) implied that the lack of fit was insignificant as compared to pure error. The CV value of a model indicates that degree of precision at which the experiments were carried out. In the present study the CV value was low (0.93) which indicated that the experiments performed were reliable. In present study the values of R^2^ and adjusted R^2^ were 0.9932 and 0.98 respectively, indicating aptness of the model. All these observations clearly showed that the model developed would be able to predict the response correctly. The adequate precision, which indicates noise to signal ratio, was found to be high (28.71) suggesting suitability of the model for navigating the design space. Further, the parity plot (Figure 1) indicated good correlation between predicted and observed responses ensuring a good fit of the model.

**Figure 2:**
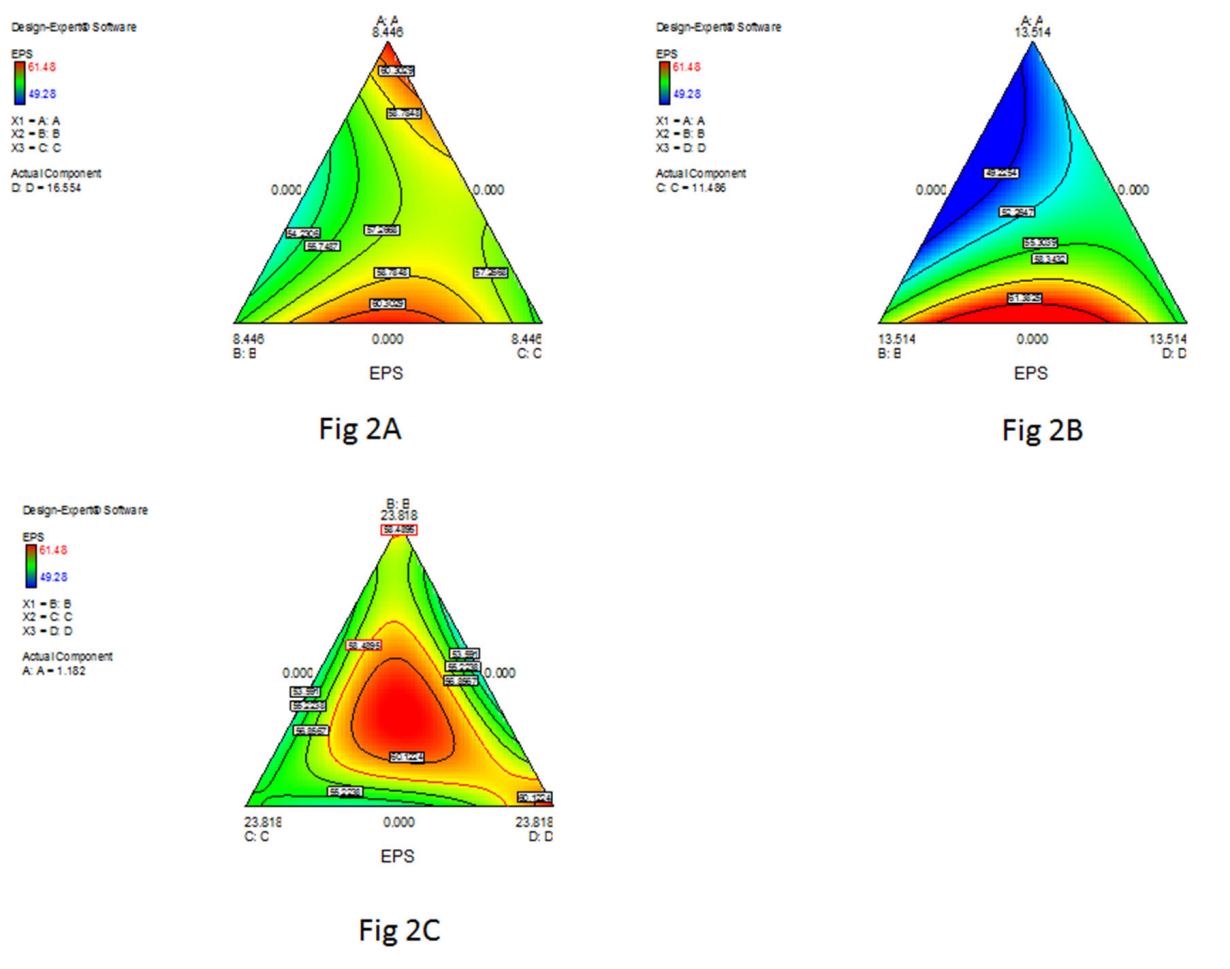
Interaction among Ethanol (A), Acetone (B) and IPA(C) at constant concentration of THF (D). **B** Interaction among Ethanol (A), Acetone (B) and THF (D) at a fixed concentration of IPA(C). **C** Interaction among Acetone (B), IPA (C) and THF (D) at a definitive concentration of Ethanol (A).

The regression equation developed is graphically represented via contour plots. These plots were used to study the effect of interaction among solvents on pullulan recovery as well as to develop optimum combination of the solvents. Three contour plots were developed considering all possible interactions. These graphical representations (Figure 2A-2C) helped us to understand the interaction, response and experimental level for each independent variable. Figure 2A indicates the effect of changing the concentration of THF at a specific combination of ethanol, acetone and IPA. Pullulan precipitation increased at higher concentration of THF and reduced as the concentration of THF was lowered. Similarly, Figure 2B represents the effect of IPA concentration while concentrations of other three solvents were kept fixed at a certain level. The results indicated that IPA has a limited effect on pullulan precipitation. Figure 2C, shows the effect of ethanol concentration while keeping other 3 solvents fixed at certain value. It is clear from the data that a lower concentration of ethanol will be more effective in combination of other selected solvents to obtain better recovery of the polymer. 

### 3: Optimization and Model validation

Stat Ease Software was used to study the interaction among different solvents as well as to develop a model to obtain an optimum combination of different solvents resulting in higher recovery of pullulan. A model was developed after performing 20 representative experiments, as suggested by design of experiment (DOE) study. The data obtained from these experiments were further utilized to develop a model to navigate the whole design space as defined by boundaries/restrictions considered while designing the experiments. This model has been used to calculate the response for all possible combinations of solvents within the design space and to predict the optimized solvent combination. The model suggested that solvent combination of A: B: C: D ratio should be maintained at 0.000:10.810:4.977:9.213 to obtain optimum pullulan recovery and predicted 64.38g/L pullulan recovery when this solvent combination is used. The experimental data showed that 65.34 (±0.21) g/L pullulan could be obtained by using the same ratio, indicating that the model is valid and can be used for understanding the effect of interaction among several solvents on pullulan recovery from cell free fermentation broth.

### 4: Effect of interactions among solvents on pullulan recovery

The obsereved responses (experimental data) and predicted responses obtained from the model are given in Table 2. It is clear from the data that pullulan recovery increased with increase in THF concentration in the solvent mixture. For example, 61.48g/L pullulan was obtained, in case of run no. 9, when only THF was used as the solvent. However,the pullulan recovery was decreased to 57.07 g/L when the concentration of THF was reduced to half in the solvent mixture (Run no. 11). Moreover, pullulan yield dropped significantly upon further decrease of THF concentration in the solvent mixture (Run no. 13). On the other hand when ethanol was used as sole solvent, only 50.53g/L pullulan was precipitated (Run no. 1). In case of Run no. 3, the decrease in ethanol concentration resulted in increase in pullulan recovery. In experimental runs (Run nos. 5, 6,7,8,9,10 ,11,16 and 17) where ethanol was omitted from the solvent mixture, pullulan recovery was higher as compared to Run no.1 , where ethanol was the only solvent used. This clearly indicated that minimal quanity of ethanol was useful for obtaining higher yield of pullulan during precipitation from fermentation broth. Acetone had also shown a positive effect in terms of yield enhancement. When the acetone concentration was doubled in the solvent mixture, the polymer precipitation was also increased (Run no. 7 and Run no.11). On the other hand, change in IPA concentration showed a limited positive effect on pullulan recovery. In case of Run no. 5 and Run no. 6, the pullulan yield differed only by 6% although , change in IPA concentration was around 2 fold. Thus, these data indicated that acetone and THF have a significant positive influence on pullulan recovery. Whereas, IPA has a limited positive effect and ethanol has a negative effect on the yield of pullulan. This was also reflected in the optimized composition of solvents obtained by using the polynomial model developed, where, ethanol concentration was the lowest and acetone and THF were used in comparable concentration ,whereas IPA was used in medium level of concentration.

## Conclusion

In the present study statistical optimization technique was used to develop a suitable model for understanding the interactions among different solvents and their effects on pullulan recovery from fermentation broth. The polynomial model developed in this study had a high F value (85.54) and R^2^ value (0.9932) indicating that the model was nicely fitted with experimental data obtained and may be used to navigate the design space to obtain an optimized solvent system for pullulan recovery from fermentation broth. The optimized solvent combination had resulted in precipitation of 65.34 (±0.21) g/L pullulan whereas the model predicted a recovery of 64.38g/L pullulan indicating that the data obtained by performing validation experiments were in well agreement with the predicted responses. 

Contrary to the earlier reports which used ethanol as the ‘solvent of choice’[5,22] , the data obtained in the present study clearly indicated that a lower concentration of ethanol in combination with other selected solvents would be better option for recovery of pullulan from fermentation broth. The optimized solvent combination had resulted in enhancing the yield of pullulan by around 7% relative to single solvent like ethanol. Hence, it may be concluded that RSM helped us to understand the interactions among different solvents and indicated that alternative solvents or combinations of solvents might be better option for pullulan recovery from cell free fermentation broth.
